# A Facile Route toward the Increase of Oxygen Content in Nanosized Zeolite by Insertion of Cerium and Fluorinated Compounds

**DOI:** 10.3390/molecules23020037

**Published:** 2018-01-24

**Authors:** Sarah Komaty, Clément Anfray, Moussa Zaarour, Hussein Awala, Valérie Ruaux, Samuel Valable, Svetlana Mintova

**Affiliations:** 1 Laboratoire Catalyse et Spectrochimie, Normandie University, ENSICAEN, UNICAEN, CNRS, 14050 Caen, France; sarah.komaty@ensicaen.fr (S.K.); moussa.zaarour@ensicaen.fr (M.Z.); hussein.awala@ensicaen.fr (H.A.); valerie.ruaux@ensicaen.fr (V.R.); 2 ISTCT/CERVOxy Group, Normandie University, UNICAEN, CEA, CNRS, 14050 Caen, France; anfray@cyceron.fr (C.A.); valable@cyceron.fr (S.V.)

**Keywords:** zeolite, nanocrystals, oxygen adsorption, FTIR, fluorinated zeolite, cerium, toxicity test

## Abstract

Enriching oxygen content within nanosized zeolite X (as synthesized Na-X) by insertion of cerium (ion exchanged Ce-X) and functionalization with bromoperfluoro-*n*-octane (fluorinated F-X) is reported. The materials were fully characterized by powder X-ray diffraction (XRD), dynamic light scattering (DLS), zeta potential, thermogravimetric analysis (TGA), nitrogen adsorption, and nuclear magnetic resonance (^19^F NMR). The O_2_ adsorption in the zeolite samples at various concentrations (0 to 165 Torr) at −196 °C was studied by in situ FTIR. The modification of nanosized zeolites did not alter their colloidal stability, crystallinity, porosity, and particle size distribution. The inclusion of cerium and bromoperfluoro-*n*-octane considerably increase the oxygen capacity by 33% for samples Ce-X and F-X in comparison to the as-synthesized Na-X zeolite. Further, toxicity tests revealed that these materials are safe, which opens the door for their implementation in medical applications, where controlled delivery of oxygen is highly desirable.

## 1. Introduction

A sufficient oxygen supply is indispensable to ensure the normal functioning of the human body. At sea level, the partial pressure of oxygen is around 21 kPa, which is enough to saturate the hemoglobin. This abundance decreases sharply with higher altitudes, reaching half of its value at 5500 m [1], and starting from 8000 m, the oxygen quantity becomes insufficient to sustain human life. Hence, ensuring adequate level of oxygen is a must for people living at high altitudes and mountain climbers. This need becomes more crucial in airplanes, where passengers are supposed to breathe at much higher altitudes (>9000 m). The storage and transportation of pure oxygen is further required for medical uses such as emergencies and anesthetic procedure where portable oxygen containers are required [2]. To fulfill these needs, several methods for the production, storage, and transportation of oxygen have been used, including the cryogenic liquid oxygen systems, oxygen pressurized cylinders, and oxygen concentrators. While the first two suffer from high cost and the need for frequent refilling, which is not always accessible, oxygen concentrators are safer [3], less expensive [4], and more practical. These systems are based on zeolite porous materials that separate oxygen from the air [2]. Despite of their superior advantages compared to readily available systems, oxygen concentrators require a constant supply of electrical energy to produce oxygen with no possibility for its storage.

For decades, zeolites have been used in a wide range of different applications. Their capability to cover the required specifications lies in their high stability, crystallinity, well-defined porous structure, and tunable hydrophobic/hydrophilic properties [5,6,7]. The ability of modifying one or more of its properties allows zeolite to fulfill the conditions required for certain applications, and hence, better performance is achieved. Classically, the modification is carried out by post-synthetic treatment including ion exchange [8,9], dealumination [10], desilication [11,12], or surface and intra-pore modification [13].

Due to the low toxicity, and in some cases small crystallite dimensions, zeolites are attracting continuous attention in the biomedical field and related applications. Furthermore, their capability in reversible adsorption/desorption of different gases such as CO, NO, and CO_2_ [14] opens the door for their use in gas storage and controlled delivery. While delivering of large amounts of such gases is often toxic, too small amounts can be ineffective. Interestingly, LTA-zeolite has shown a remarkable success in long term storage of NO (tested up to one year) without losing the deliverable capacity. Additionally, a controlled diffusion kinetics was achieved in the absence of side effects that can be encountered by chemically produced NO from acidified nitrite creams [15]. Oxygen dissolving by functionalized zeolites has also been reported [16]. However, the described systems were too large to be used in medical applications at the cellular level. 

Moreover, zeolite X (FAU-type framework structure) with a large supercage (1.1 nm) is used as gas carrier [17] and drug delivery system [18,19]. Recently, the preparation and modification of FAU [20] and EMT [21] types zeolite nanocrystals with anti-microbial properties associated with toxic effects on various eukaryotic cell lines were reported.

Herein, we report the functionalization of zeolite X nanocrystals (Na-X) by bromoperfluoro-*n-*octane (F-X) or cerium cation (Ce-X) as a method to increase the oxygen content to be further used in oxygen storage and delivery. In fact, perfluorinated compounds (PFCs) have been widely used in biochemistry, pharmacology, and biotechnology and most importantly in biomedical field as gas carriers due to the solubility of respiratory gases in these compounds being almost twice that of their hydrocarbon counterparts [22]. Additionally, this class of compounds is known to be inert [23] with low surface tension and capable of rapidly and extensively liberating dissolved oxygen when needed [24]. All these features make PFCs attractive solvents for in vivo oxygen delivery. Among several hundreds of fluorinated compounds have been examined, very few had the pertinent physicochemical and biological criteria for in vivo oxygen delivery such as rapid excretion, high purity and absence of clinically significant side effect. One of the best candidates that achieve these requirements is bromoperfluoro-*n*-octane [23,25]. This compound presents rapid excretion rate and absence of side effects of this product or metabolic sub-products [25].

On the other hand, cerium-based materials such as ceria (CeO_2_) is known for its strong oxygen storage capabilities [26,27,28]. In oxygen-deficient conditions, it acts as an oxygen donor and transforms to Ce_2_O_3_, while in the presence of sufficient quantities of oxygen, the Ce_2_O_3_ is re-oxidized and becomes ready for a second cycle of delivery. Furthermore, the combination of zeolite, more specifically zeolite X with CeO_2_, was used to increase the efficiency of adsorption; the dispersion of CeO_2_ on the zeolite increases the number of adsorption active sites, while the zeolite acts as an active matrix with acid sites that are further active for adsorption [26].

In this article, we investigate the oxygen adsorption capacity of as synthesized nanosized zeolite X (Na-X) subjected to modification by insertion of cerium or fluorinated organic compound. The incorporation of Ce in zeolite X (sample Ce-X) was proved by chemical analysis, whereas ^19^F NMR together with TGA gave a clear evidence of the incorporation bromoperfluoro-*n*-octane (sample F-X). All zeolites were fully characterized by XRD, DLS, zeta potential and FTIR. A preservation of the crystalline structure and colloidal stability after modification is presented, while the oxygen sorption capacity Ce-X and F-X was enhanced by 33%. Additionally, the cytotoxicity tests were performed, and no toxicity of zeolites was observed, which opens the door for future medical uses of zeolites with a great capacity for oxygen.

## 2. Materials and Methods

### 2.1. Preparation of Nanosized Zeolite

Materials: Al powder (325 mesh, 99.5%, Alfa Aesar (Karlsruhe, Germany), NaOH (97%, Sigma-Aldrich (Lyon, France), SiO_2_ (Ludox-HS 30, 30 wt% SiO_2_, pH = 9.8, Sigma-Aldrich), sodium hydroxide (98.7%, prolabo), sodium silicate (25.5–26.5% SiO_2_, 7.5–8.5% Na_2_O, prolabo), sodium aluminate (50–56% Al_2_O_3_; 40–45% Na_2_O, Sigma-Aldrich), bromoperfluoro-*n-*octane (Sigma-Aldrich), CeCl_3_∙5H_2_O (Sigma-Aldrich).

Preparation of zeolite X nanocrystals (sample Na-X): Stable suspension of discrete nanosized zeolite (Na-X framework zeolite type) is prepared from colloidal precursor suspension under hydrothermal condition according to a published procedure [29].

Fluorination of zeolite X nanocrystals (sample F-X): 3 mL of Na-X suspension (3 wt% in H_2_O) was mixed with 1 mL of bromoperfluoro-*n-*octane solution (50 wt% in acetone) in a polypropylene bottle. The mixture was then kept under stirring at 40 °C for 2 h and washed by acetone. This procedure was repeated three times, and the final product was washed two times with acetone to remove the excess of fluorinated compound prior the final washing with water.

Modification of X nanocrystals by cerium (sample Ce-X): 25 mL of CeCl_3_∙5H_2_O (3 mM in H_2_O) were mixed with 5 mL of Na-X suspension (2.5 wt% in H_2_O) and kept under stirring at room temperature for 1 h. The resulting product was then washed by double distilled water. The same procedure was repeated three times. 

### 2.2. Characterization of Nanosized Zeolite

X-ray diffraction (XRD) characterization: The crystallinity of the zeolite powders (Na-X, F-X, and Ce-X) was studied by XRD analyses carried out with *PANalytical* X’Pert Pro diffractometer with CuKα monochromatized radiation (λ = 1.5418 Å).

Dynamic light scattering (DLS) and zeta potential analyses: The size of the nanoparticles was measured by a Malvern Zetasizer Nano instrument using a backscattering geometry (scattering angle of 173°, He-Ne laser with a 3 mW output power at a wavelength of 632.8 nm). The DLS analyses were performed on samples with a solid concentration of 2 wt%. The surface charge of the crystals was determined by measuring the zeta potential value of suspensions at a constant solid concentration (2 wt%) and pH = 8.5.

Nitrogen adsorption analysis: The porosity of the samples was measured using a Micrometrics ASAP 2020 volumetric adsorption analyzer. Samples were degassed at 275 °C under vacuum overnight prior to the measurement. The external surface area and micropore volume were estimated by alpha-plot method using Silica-1000 (22.1 m^2^∙g^−1^ assumed) as a reference. The micropore and mesopore size distributions of samples were estimated by the Nonlocal Density Functional Theory (NLDFT) and Barret-Joyner-Halenda (BJH), respectively using the desorption branch of the isotherm.

Thermogravimetric analysis (TGA): The moisture content and the organic compound (bromoperfluoro-*n*-octane, present in F-X sample) were investigated using a SETSYS (SETARAM, Caluire, France) analyzer (heating rate of 5 °C∙min^−1^ under 40 mL∙min^−1^ flow of air).

Liquid state ^19^F NMR spectroscopy: Liquid State NMR spectra of zeolite suspensions were recorded in deuterated solvents (F-X zeolite in D_2_O) on a 500 MHz Bruker Avance III (Karlsruhe, Germany) apparatus. The chemical shifts are calibrated to residual proton resonance of CFCl_3_ (δH 0 ppm).

Oxygen adsorption study by in situ FTIR: Zeolite powders (samples Na-X, F-X, and Ce-X) were pressed (~10^7^ Pa) into self-supported disks (2 cm^2^ area, 20 mg∙cm^−2^) and placed in an IR cell equipped with KBr windows. IR spectra were recorded using a Nicolet) 6700 IR spectrometer (Thermo Scientific, Villebon sur Yvette, France) equipped with a mercury cadmium telluride (MCT) detector and an extended KBr beam splitter. Spectra were recorded in the 400−5500 cm^−1^ range at 4 cm^−1^ with 128 scans. A homemade cell that can be evacuated or flooded with different gases and also heated up to 600 °C or cooled down to −196 °C was used. The activation of the samples was performed in situ at 250 °C for 2 h under vacuum prior the measurements. Various amounts of oxygen (0–165 Torr) were introduced into the cell and kept in equilibrium for 5 min before recording each spectrum. To allow the comparison of different samples, all the spectra were normalized to the samples’ mass and plotted as absorbance per gram over the wavelength.

Chemical analysis: The chemical composition of the FAU samples was determined by inductively coupled plasma (ICP) optical emission spectroscopy using a Varian ICP-OES 720 ES. The chemical composition (in wt%) for the samples was found as follows: Na-X: Si (18.1), Al (14.6), Na (13.4) and Ce-X: Si (17.6), Al (14.3), Na (12.0), Ce (1.2).

### 2.3. Toxicity Tests of Nanosized Zeolites

Cell lines: A human glioblastoma cell lines, U87-MG purchased from American Type Culture Collections (ATCC, Manassas, VA, USA) (ATCC) and HEK 293 cells (Human Embryonic Kidney cells) were used. Cells were cultured in DMEM (Sigma-Aldrich, Saint-Quentin Fallavier, France) supplemented with 10% fetal bovine serum (Eurobio, Courtaboeuf, France), 2 mM glutamine (Sigma-Aldrich) and 100 U/mL penicillin/streptomycin (Sigma-Aldrich). Cells were seeded in 24-wells plates at a concentration of 5 × 10^4^ cells/mL and maintained in culture at 37 °C with 5% CO_2_ and 95% humidity.

Primary culture of astrocytes: Cerebral cortices were isolated from neonatal (1 to 3-day-old) Swiss mice (CURB, Caen, France) carefully stripped of the meninges and dissociated to generate a single-cell suspension. Cultures were allowed to grow in a humidified 5% CO_2_ incubator at 37 °C to confluence (15–20 days) prior to use in DMEM supplemented with 10% fetal bovine serum (Eurobio), 10% horse serum (Eurobio), 2 mM glutamine (Sigma-Aldrich), and 100 U/mL penicillin/streptomycin (Sigma-Aldrich). At about 80% confluence, the growth medium was replaced by the same medium.

Cells treatment: Cells were exposed to zeolite suspensions (Na-X, F-X, and Ce-X) for 24 or 48 h. Zeolites were diluted in culture medium at a concentration of 50, 100, or 400 µg/mL and added directly in the wells.

Cells viability: Cell viability was analyzed after 24 h or 48 h exposure to zeolites (Na-X, F-X, and Ce-X) with the WST-1 assay (Roche) according to manufacturer’s instructions.

Bacterial culture: *E. coli* (Life Technology) glycerol stock (−80 °C) was thawed and cultivated in 10 mL of liquid Luria Broth (LB) culture medium (Sigma-Aldrich) overnight at 37 °C on a shaker (220 rpm). Then, samples were transferred into fresh LB medium containing different concentrations of zeolite nanocrystals (0, 50, 100, and 400 µg/mL), distributed in a 96 wells plate (100 µL per well), and incubated at 37 °C on a shaker (220 rpm) for 3.5 h. Bacterial growth was assessed by measuring the optical density at 600 nm (OD_600_) every 15 min using a spectrophotometer (Asys UVM 340, Biochrom Ltd., Cambridge, UK). Fresh LB medium containing different concentrations of zeolites were used as blank samples.

Statistical analyses: Data are presented as mean ± SD. Statistical analyses were obtained with a JMP programs (SAS Institute, Cary, NC, USA).

## 3. Results and Discussion

### 3.1. Structural Characterizations

**Figure 1:**
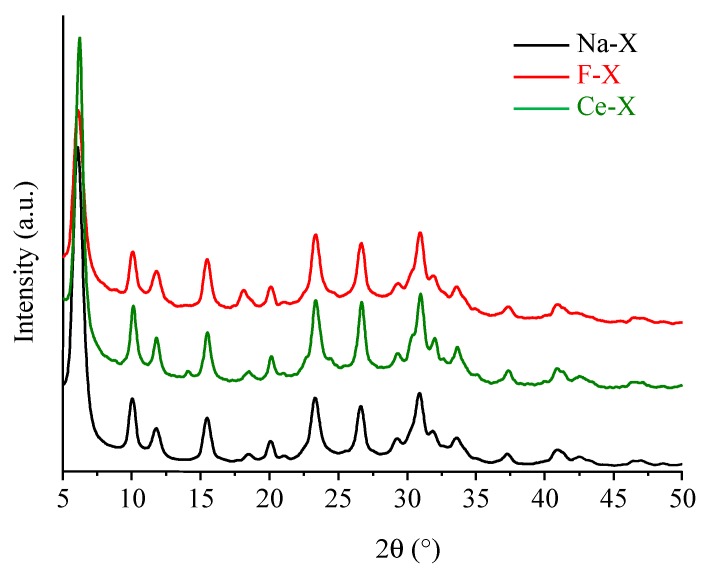
X-ray diffraction (XRD) patterns of as-synthesized Na-X, fluorinated F-X, and cerium ion exchanged Ce-X zeolites.

The high crystallinity of zeolite nanocrystals is preserved after modification, i.e., the X-ray diffraction patterns of as-synthesized Na-X zeolite as well as the functionalized F-X and ion-exchanged Ce-X show a very similar profile with broadened peaks reflecting the small particle size of the crystallites. The typical Bragg peaks corresponding to the FAU-type zeolite are present in the XRD patterns depicted in Figure 1.

**Figure 2:**
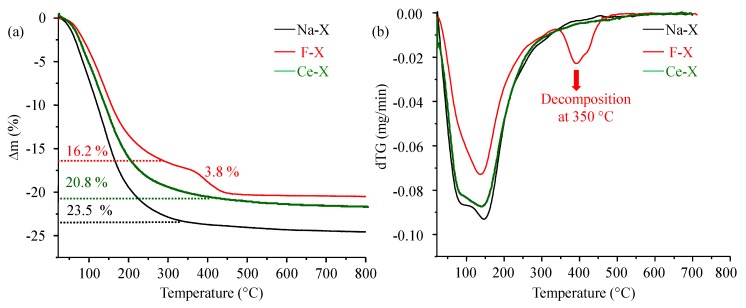
(**a**) Thermogravimetric (TG) and (**b**) differential thermogravimetric (DTG) curves of as-synthesized Na-X, fluorinated F-X, and cerium ion exchanged Ce-X zeolites.

Despite the absence of remarkable variations among the XRD patterns, distinct TG profiles were recorded for the three samples (Figure 2). Samples Na-X, Ce-X, and F-X show a common mass loss at 150 °C corresponding to water adsorbed on the surface and in the channel of the nanoparticles contributing to 23.5%, 20.8%, and 16.2% of their total masses, respectively. While Na-X and Ce-X present one single mass loss, the F-X possesses a second significant mass loss (3.8%) at 350 °C that corresponds to the release of the bromoperfluoro-*n-*octane. Following a simultaneous mass spectroscopy of species emerging in gas phase, these species are attributed to the dissociation products of the perfluorinated organic compound used for the modification. This finding is further supported by ^19^F NMR spectrum containing sharp and intense peaks corresponding to a fluorinated organic species (Figure S1). Regardless of the solvent effect, the position and the number of these peaks resemble those of bromoperfluoro-*n*-octane (Figure S2) thus assuming that the compound was adsorbed in its molecular form.

**Figure 3:**
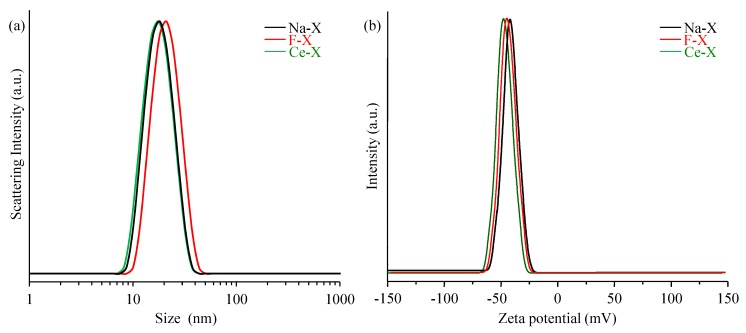
(**a**) Dynamic light scattering (DLS) and (**b**) zeta potential curves of as-synthesized Na-X, fluorinated zeolite F-X, and cerium ion exchanged Ce-X zeolites.

DLS measurements of the Na-X suspension show uniform particles of crystals with an average size of 18 nm (Figure 3a). This homogeneity is retained after functionalization. Uniform particles of 18 nm and 20 nm for Ce-X and F-X, respectively, were detected. In all the three cases a narrow and monomodal particle size distribution with a polydispersity index (PDI) of around 0.1 was recorded.

The stability of the zeolite nanocrystals in colloidal suspensions was further studied using zeta potential measurements (Figure 3b). The values are found to vary from −45 mv (Na-X) to −44 mv (Ce-X) to −39 mV (F-X), thus illustrating the highly stability of all three colloid suspensions.

**Figure 4:**
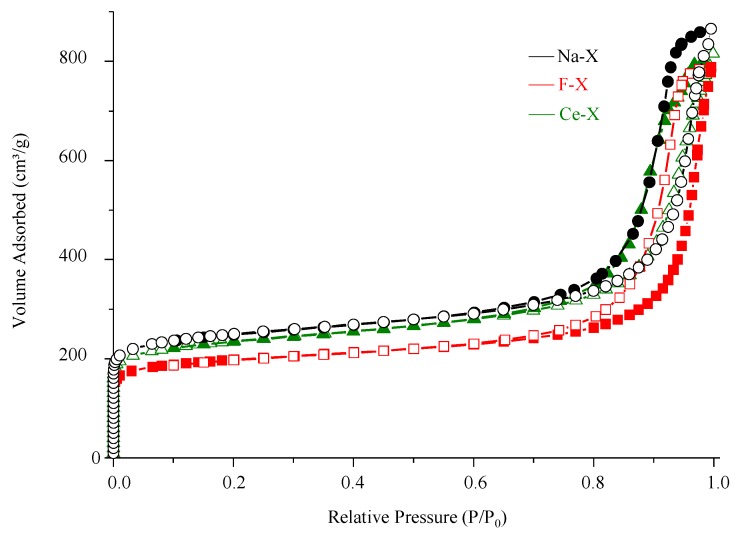
N_2_ adsorption (full symbols) and desorption (empty symbols) isotherms of as-synthesized Na-X (black circle), fluorinated F-X (red square), and cerium ion exchanged Ce-X (green triangle) zeolites.

The porosity of zeolite nanoparticles before and after functionalization was investigated by N_2_ sorption analysis (Figure 4, Table 1). All samples exhibit Type I isotherm at low P/P_0_, which is characteristic for microporous materials. A high adsorption uptake for the three samples at P/P_0_ > 0.8 is due to the high textural mesoporosity resulting from the closely packed zeolite nanoparticles with similar particle sizes. Furthermore, a decrease of the BET surface area from 880 m^2^∙g^−1^ (Na-X) to 825 m^2^∙g^−1^ (F-X) suggests a partial modification of the external surface and pores with bromoperfluoro-*n*-octane. In contrast, the Ce-X showed a similar BET surface to the as synthesized Na-X.

### 3.2. Oxygen Adsorption Capacity of Nanosized Zeolites: In Situ IR Study

**Figure 5:**
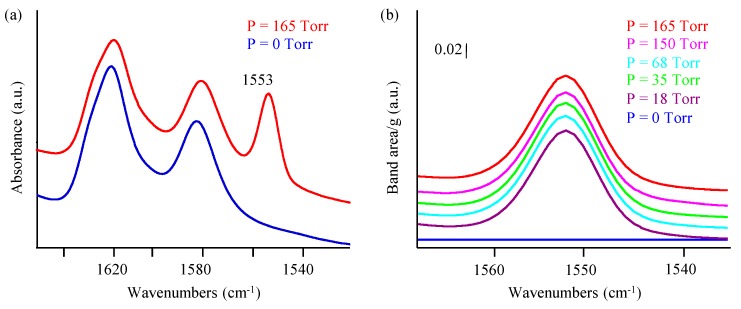
(**a**) IR spectra of as synthesized Na-X at 0 and 165 Torr O_2_ and (**b**) evolution of IR band at 1553 cm^−1^ corresponding to O_2_ adsorbed on the Na-X at different pressures (T = −196 °C).

Upon its adsorption on zeolite, the IR silent O–O stretching mode of molecular oxygen becomes active at 1556 cm^−1^ [6,30,31,32]. The prepared zeolites show a band centered at 1553 cm^−1^ (Figure 5, Figures S3 and S4) which is in the same region of the reported values at 1553 and 1557 cm^−1^ (shoulder) corresponding to O_2_ adsorbed on the Brønsted and on the Na sites, respectively [6,33]. In the current case, a broad band extends from 1540 to1565 cm^−1^ masks the shoulder at 1557 cm^−1^, and it appears and then disappears repetitively after several cycles of oxygen adsorption/desorption cycles. No remarkable change in the intensity of this band is observed, thus confirming the stability of the sample based on several sorption cycles without losing its efficiency.

On the other hand, the small shift of the band at 1553 cm^−1^ compared to free oxygen reflects a weak bonding [31], which is further supported by the appearance of the band only at −196 °C, while it is not detected at room temperature.

The adsorption of oxygen further induced some modification in the OH stretching vibration region [30]. The silanol band located at 3740 cm^−1^ for Ce-X [34] is gradually shifted to 3733 cm^−1^ due to the perturbation promoted by oxygen favoring the formation of hydrogen bonded species (Figure S5). The partially overlapping bands at 3680–3670 cm^−1^ and 3650–3620 cm^−1^ are assigned to Al-OH [34] and Brønsted OH groups [30,35] in the supercage [34], respectively. The evolution of bands of the different species after being in contact with oxygen allows judging the strength of their interactions with O_2_ as well as the homogeneity of these sites. For instance, the isolated silanols weakly interact with oxygen present a shift of −7 cm^−1^. Furthermore, the very small broadening of the band after O_2_ uptake reflects the homogeneity of the silanols. In contrast, the Brønsted sites are highly influenced by the oxygen adsorption evidenced by a strong shift of 40 cm^−1^, while the large broadening recorded can be explained by the non-equal interaction of oxygen with different Al sites [31].

**Figure 6:**
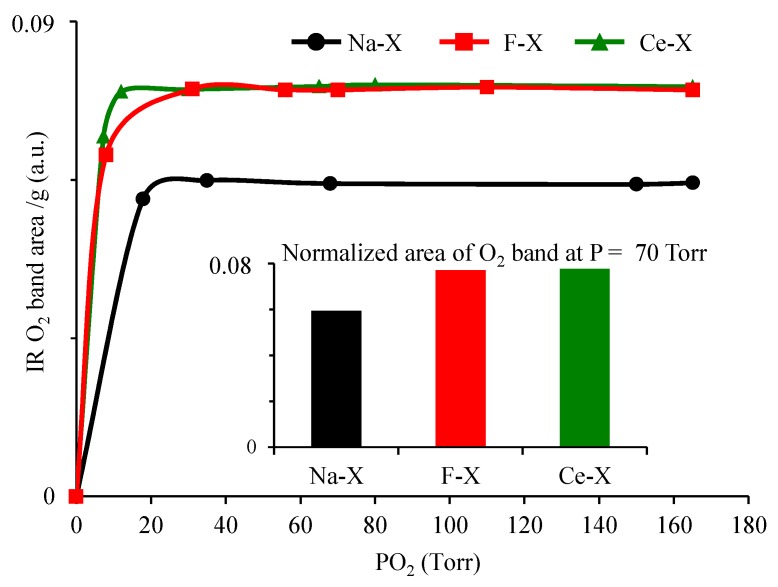
Evolution of O_2_ band area of as-synthesized Na-X, fluorinated F-X, and cerium ion exchanged Ce-X zeolites versus applied oxygen pressure at −196 °C. Inset: normalized area of oxygen band upon adsorbing 70 Torr of oxygen.

The modification of zeolite by introducing Ce or fluorinated organic compound enhances the kinetics as well as the efficiency of oxygen adsorption. A faster loading of oxygen is revealed by the higher slope of the curve presenting the area of oxygen versus pressure introduced (Figure 6). An increase of 33% in the loading capacity for F-X and Ce-X compared to Na-X is achieved. This increase is attributed to the availability of additional amount of oxygen dissolved by the associated fluorinated compound or induced by the presence of cerium cation. Hence Ce-X and F-X encounter two types of oxygen: (i) those adsorbed directly on the zeolite after applying oxygen pressure and (ii) those dissolved by the fluorinated compound or associated to cerium that constitutes an additional pool of oxygen.

It is worth mentioning that all the three zeolites showed long-term storage capacity of oxygen (tested up to 2 h). The amount of oxygen adsorbed stayed constant with no pronounceable decrease upon maintaining the sample in an isolated environment.

Owing to their ultra-small dimension, high oxygen storage capacity, and fast release kinetics, these materials can be considered for medical applications (oxygen storage and delivery). Hence, a detailed study of their toxicological effect was considered. 

### 3.3. Toxicity Tests

**Figure 7:**
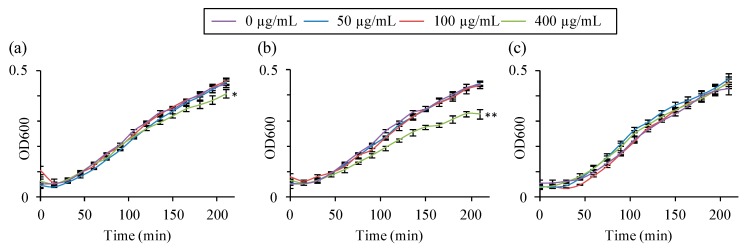
Quantification of the growth of *E. coli* bacteria during 3.5 h of exposure to increasing concentrations of (**a**) as-synthesized Na-X; (**b**) fluorinated F-X; and (**c**) cerium ion exchanged Ce-X zeolites. Bacterial growth was assessed by measuring the optical density (OD_600_). Mean ± SD, *n* = 3. HSD Tuckey post-hoc test after a significant analysis of variance (ANOVA) (** *p* < 0.0001; * *p* < 0.01 vs. control group (0 µg/mL)).

To verify that the Ce cation and the fluorinated compound associated with the zeolite did not induce an in vitro cell damage, the viability of bacteria and multiple cell types originating from various organs exposed to increasing concentration of zeolites was measured. No changes in the growth of *E. coli* was observed in the presence of zeolites Na-X and Ce-X up to 100 µg/mL or F-X up to 400 µg/mL for (Figure 7).

**Figure 8:**
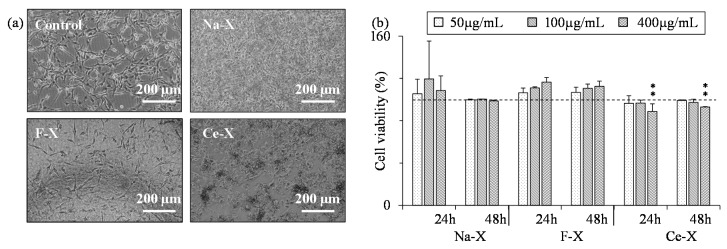
(**a**) Representative photographs (phase contrast) of U87-MG cells after 48 h exposure to 400 µg/mL of as-synthesized Na-X, fluorinated F-X, cerium ion exchanged Ce-X zeolites, and a control sample (H_2_O); (**b**) Quantification of U87-MG cells viability after 24 h and 48 h exposure to Na-X, F-X, and Ce-X zeolites with different concentrations. Cell viability was assessed using the WST-1 test. Mean ± SD. *n* = 3; HSD Tuckey post-hoc test after a significant ANOVA (** *p* < 0.005 vs. control group).

**Figure 9:**
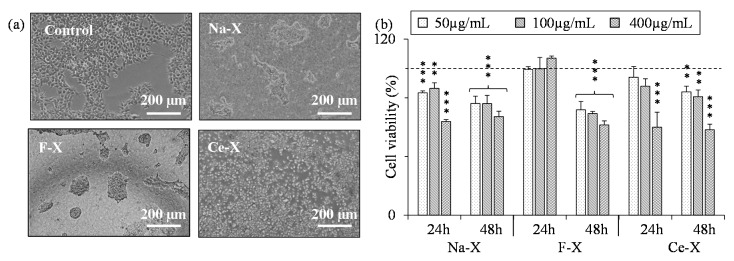
(**a**) Representative photographs (phase contrast) of HEK 293T cells after 48 h exposure to 400 µg/mL of as-synthesized Na-X, fluorinated F-X, the cerium ion exchanged Ce-X zeolites, and a control sample (H_2_O); (**b**) Quantification of HEK 293T cells viability after 24 h and 48 h exposure to Na-X, F-X, and Ce-X zeolites with different concentrations. Cell viability was assessed using the WST-1 test. Mean ± SD. *n* = 3; HSD Tuckey post-hoc test after a significant ANOVA (* *p* < 0.05; ** *p* < 0.005; *** *p* < 0.0005 vs. control group).

Additionally, a human glioblastoma cell line (U87-MG) and a human kidney cancer cell line (HEK293), both chosen to have a marked proliferation rate in vitro were exposed to zeolites with various concentrations. While the former showed no evidence of toxicity in the presence of Na-X and F-X with concentration up to 400 µg/mL after 24 h or 48 h of exposure, the Ce-X induced a small, yet significant decrease in cell viability at 400 µg/mL (Figure 8). On the other hand, HEK293 cells showed higher sensitivity to the zeolites: a notable decrease in the cell viability was observed upon 48 h of exposure to 50 µg/mL of Na-X, Ce-X, and F-X (Figure 9).

**Figure 10:**
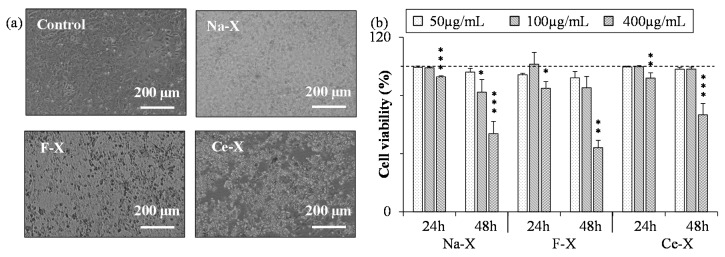
(**a**) Representative photographs (phase contrast) of astrocytes after 48 h exposure to 400 µg/mL of as-synthesized Na-X, fluorinated F-X, the cerium ion exchanged Ce-X zeolites, and a control sample (H_2_O); (**b**) Quantification of astrocytes viability after 24 h and 48 h exposure to Na-X, F-X, and Ce-X zeolites with different concentrations. Cell viability was assessed using the WST-1 test. Mean ± SD. *n* = 3; HSD Tuckey post-hoc test after a significant ANOVA (* *p* < 0.05; ** *p* < 0.005 ; *** *p* < 0.0005 vs. control group).

Finally, the effect of zeolites on the viability of a primary culture of mice astrocytes, low proliferating cells, was investigated (Figure 10). No influence on the cell survival was induced by the addition of Na-X, F-X, and Ce-X zeolites with concentration up to 100 µg/mL. In contrast, after 48 h exposure to the highest zeolite concentration (400 µg/mL), a decrease in cell survival down to 45% was observed.

To summarize our results, the half maximal inhibitory concentration (IC50), which represents the concentration required to decrease cell viability by 50%, was calculated for the three cell lines investigated in the presence of Na-X, F-X, and Ce-X zeolites (Table 2).

## 4. Conclusions

An efficient method for enriching the oxygen content within nanosized zeolite X (Na-X) is reported through its association with bromoperfluoro-*n*-octane (F-X) or cerium cation (Ce-X). The performed modifications allowed an increase of the oxygen adsorption capacity by 33% for F-X and Ce-X compared to Na-X without influencing zeolite crystallinity, structure, or size. The reason behind these improvements lies in the high oxygen dissolving/association capabilities of bromoperfluoro-*n*-octane and cerium cations, which permits supplying the zeolite with an additional pool of oxygen. The modified zeolites show very low toxicity on bacteria, mice astrocytes, and human cancer cells, thus opening the door to their testing and use in medical field as oxygen carriers.

## Figures and Tables

**Table 1:** BET-specific surface area (S_BET_), micropore volume (V_mic_) and total pore volume (V_total_) of as synthesized Na-X, fluorinated F-X, and ion exchanged Ce-X zeolites.

	S_BET_ (m^2^∙g^−1^)	V_mic_ (cm^3^∙g^−1^)	V_total_ (cm^3^∙g^−1^)
Na-X	880	0.30	1.40
F-X	825	0.27	1.28
Ce-X	875	0.28	1.35

**Table 2:** Half maximal inhibitory concentration (IC50) values (µg/mL) for three cell lines (Astrocytes, HEK 293T, U87-MG) in the presence of Na-X, F-X, and Ce-X zeolites.

Zeolite	Astrocytes		HEK 293T		U87-MG	
	24 h	48 h	24 h	48 h	24 h	48 h
Na-X	N/A	422 ± 100	569 ± 61	768 ± 210	N/A	N/A
F-X	N/A	359 ± 34	N/A	528 ± 75	N/A	N/A
Ce-X	N/A	622 ± 141	504 ± 71	474 ± 41	N/A	N/A

## Supplementary Materials

The supplementary materials are available online.
