# Supplementary material for: A Facile Route toward the Increase of Oxygen Content in Nanosized Zeolite by Insertion of Cerium and Fluorinated Compounds
Source: Molecules. 2018 Jan 24;23(2):37. doi: 10.3390/molecules23020037 (PMC6017005; doi:10.3390/molecules23020037)
Supplement: Supplementary file 1 [file molecules-23-00037-s001.pdf]

Supporting information

A facile route toward the increase of oxygen content in nanosized zeolite by insertion of cerium and fluorinated compounds

Sarah Komaty,[a] Clément Anfray,[b, §] Moussa Zaarour,[a, §] Hussein Awala,[a] Valerie Ruaux,[a] Samuel Valable,[b] and Svetlana Mintova[a,*]

[a] Laboratoire Catalyse et Spectrochimie, Normandie University, ENSICAEN, UNICAEN, CNRS, 14050 Caen, France.

[b] ISTCT/CERVOxy group, Normandie University, UNICAEN, CEA, CNRS, 14050 Caen, France.

§: Both authors contributed equally to this work.

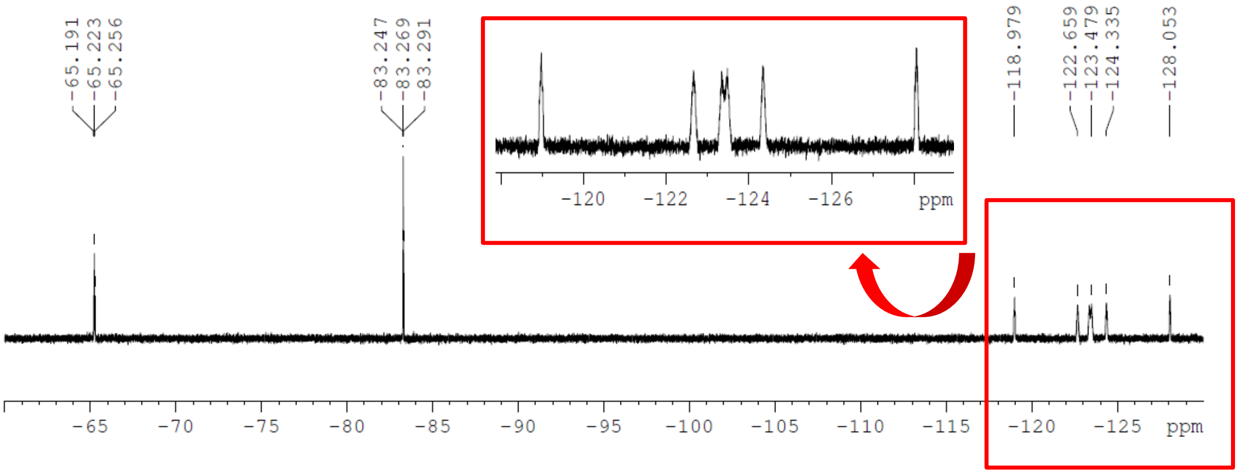

Figure S1. ^{19}F NMR spectrum of fluorinated zeolite suspension (F-X in D_2O).

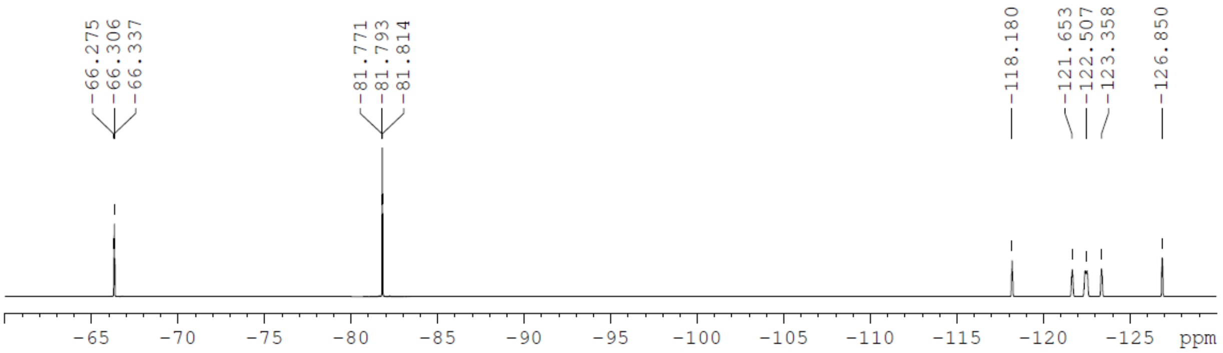

Figure S2. ^{19}F NMR spectrum of pure bromoperfluoro-n-octane in $(CD_3)_2CO$.

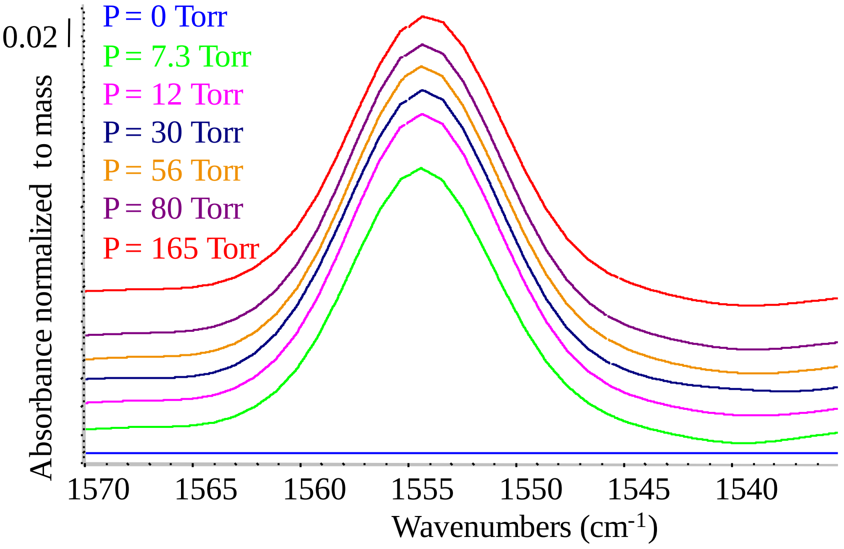

Figure S3. Evolution of IR band at 1553 cm^{-1} corresponding to O_2 adsorbed on Ce-X at different pressures (T = -196 ℃).

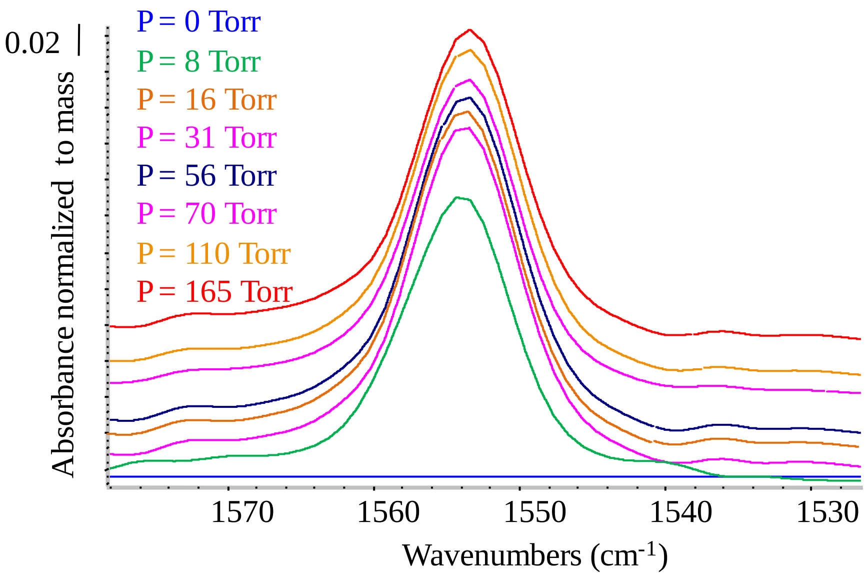

Figure S4. Evolution of IR band at 1553 cm^{-1} corresponding to O_2 adsorbed on F-X at different pressures (T = -196 ℃).

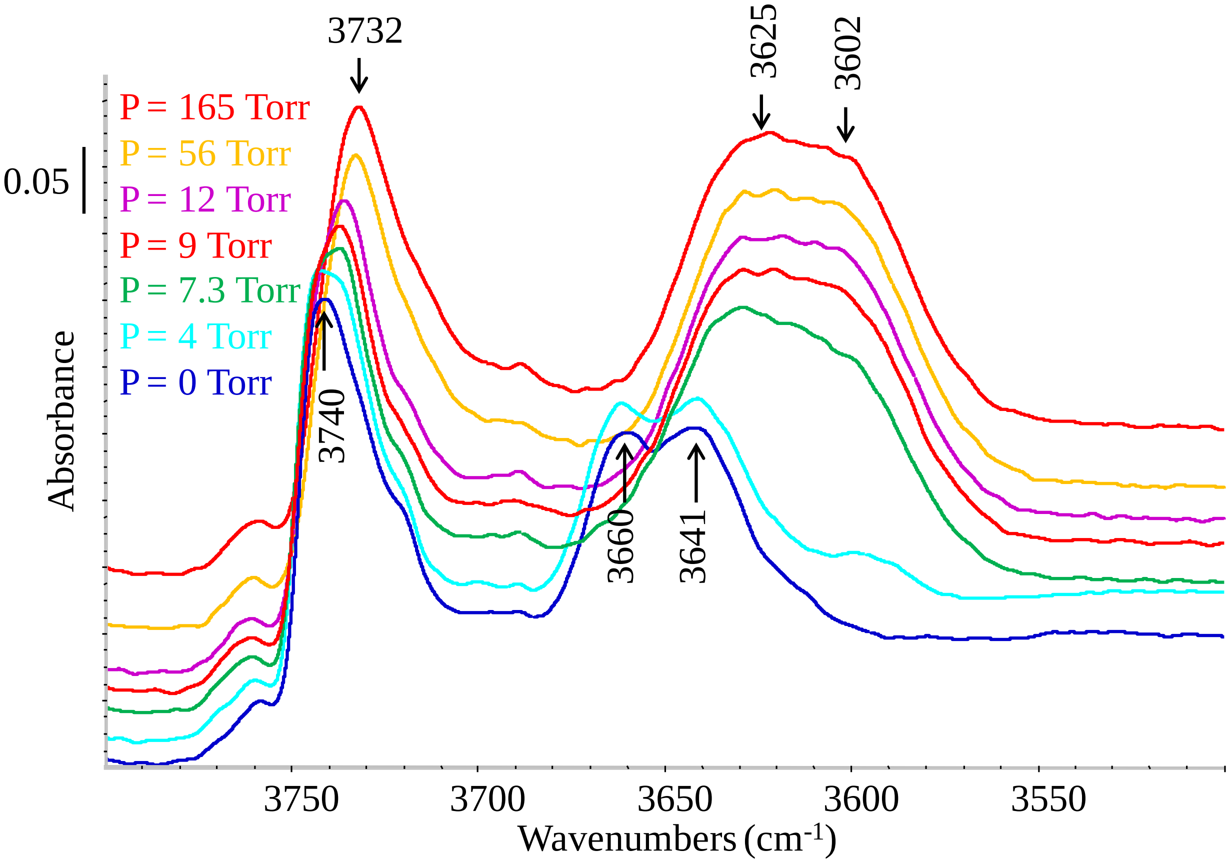

Figure S5. IR spectra of Ce-X upon oxygen adsorption at different pressures (T = -196 ℃).
